# The *Staphylococcus aureus* ArlRS Two-Component System Is a Novel Regulator of Agglutination and Pathogenesis

**DOI:** 10.1371/journal.ppat.1003819

**Published:** 2013-12-19

**Authors:** Jennifer N. Walker, Heidi A. Crosby, Adam R. Spaulding, Wilmara Salgado-Pabón, Cheryl L. Malone, Carolyn B. Rosenthal, Patrick M. Schlievert, Jeffrey M. Boyd, Alexander R. Horswill

**Affiliations:** 1 Department of Microbiology, Roy J. and Lucille A. Carver College of Medicine, University of Iowa, Iowa City, Iowa, United States of America; 2 Department of Biochemistry and Microbiology, Rutgers University, New Brunswick, New Jersey, United States of America; University of Tubingen, Germany

## Abstract

*Staphylococcus aureus* is a prominent bacterial pathogen that is known to agglutinate in the presence of human plasma to form stable clumps. There is increasing evidence that agglutination aids *S. aureus* pathogenesis, but the mechanisms of this process remain to be fully elucidated. To better define this process, we developed both tube based and flow cytometry methods to monitor clumping in the presence of extracellular matrix proteins. We discovered that the ArlRS two-component system regulates the agglutination mechanism during exposure to human plasma or fibrinogen. Using divergent *S. aureus* strains, we demonstrated that *arlRS* mutants are unable to agglutinate, and this phenotype can be complemented. We found that the *ebh* gene, encoding the Giant Staphylococcal Surface Protein (GSSP), was up-regulated in an *arlRS* mutant. By introducing an *ebh* complete deletion into an *arlRS* mutant, agglutination was restored. To assess whether GSSP is the primary effector, a constitutive promoter was inserted upstream of the *ebh* gene on the chromosome in a wildtype strain, which prevented clump formation and demonstrated that GSSP has a negative impact on the agglutination mechanism. Due to the parallels of agglutination with infective endocarditis development, we assessed the phenotype of an *arlRS* mutant in a rabbit combined model of sepsis and endocarditis. In this model the *arlRS* mutant displayed a large defect in vegetation formation and pathogenesis, and this phenotype was partially restored by removing GSSP. Altogether, we have discovered that the ArlRS system controls a novel mechanism through which *S. aureus* regulates agglutination and pathogenesis.

## Author Summary

*Staphylococcus aureus* is a bacterial pathogen that is responsible for causing significant disease in humans. The development of antibiotic resistant strains has made these infections more difficult to treat, and an improved understanding of how this pathogen causes infections will facilitate the development of new tools for treatment. It has long been recognized that *S. aureus* can bind human matrix proteins to form stable clumps in a process called agglutination, but the importance of agglutination during infection is only just becoming understood. In this work, we developed several techniques to investigate the *S. aureus* agglutination mechanism. We discovered that the ArlRS two-component regulatory system controls agglutination by regulating the expression of the *ebh* gene, which encodes the Giant Staphylococcal Surface Protein (GSSP). When ArlRS is non-functional, *S. aureus* agglutination is prevented through the action of GSSP. These phenotypes were confirmed in a rabbit model of sepsis and infective endocarditis, demonstrating that ArlRS is an important regulator of virulence. Taken together, the identification of ArlRS as a regulator of *S. aureus* agglutination and pathogenesis may lead to innovative directions for therapeutic development.

## Introduction

*Staphylococcus aureus* is a Gram-positive opportunistic pathogen that exists as part of the normal human microflora in approximately one third of the human population [1]. This pathogen is responsible for causing a wide range of acute and chronic infections resulting in significant morbidity and mortality in both the hospital and community settings. Further, the spread of methicillin-resistant *S. aureus* into the community setting (CA-MRSA) and the severe disease associated with these infections has led to its emergence as a major public health problem [2], [3]. The success of *S. aureus* as a pathogen arises in part from its genetic malleability, enabling the rapid acquisition of antimicrobial resistance mechanisms and advantageous virulence factors, and the diverse signal transduction mechanisms that can respond to both environmental and host cues [4].

*S. aureus* is the single most frequent cause of healthcare-associated infections, many of which are persistent and remain recalcitrant to treatment [2]. These types of infections are typically chronic, such as endocarditis, osteomyelitis, and implant-associated infections, and have been linked to the organism's ability to form biofilms [5], [6]. Biofilms can be defined as a community of cells encased in a protective matrix containing eDNA, proteins, and polysaccharides that are attached to a surface. This survival mechanism provides an increased capacity for bacteria to persist in hostile environments created by exposure to antibiotics and host immune defenses [5], [7], [8].

Recently, studies evaluating chronic infections with *Pseudomonas aeruginosa* suggested this pathogen predominates in cellular aggregates, as opposed to existing as surface-attached biofilms [9]–[11]. Upon further investigation, these aggregates were shown to perform a similar function as biofilms by maintaining resistance properties to antibiotics and host defenses. Additionally, *P. aeruginosa* aggregates consisted of a variety of matrix components commonly associated with biofilm formation, including eDNA and polysaccharides [10]. However, investigation of aggregate composition of another pathogen, *S. aureus*, revealed important differences between biofilm and aggregates. *S. aureus* aggregates are predominately composed of polysaccharide and display increased metabolic activity as opposed to typical bacterial biofilms and *P. aeruginosa* aggregates [10], [12]. However, it is difficult to predict how the identified properties of aggregates relate to pathogenesis since *S. aureus* exploits host proteins for agglutination *in vivo* [13], [14]. *S. aureus* agglutination is a process through which cells bind matrix proteins and form stable clumps that aid evasion of host defenses and establishment of infection. While some initial studies have been performed, the mechanisms through which *S. aureus* controls agglutination remain largely undefined.

This study investigates the mechanisms required for *S. aureus* agglutination *in vitro* and defines the two-component system, ArlRS, as a novel regulator of clumping and pathogenesis. Fournier and Hooper initially identified and characterized ArlRS as a regulator of autolysis [15]. However, recent studies by Memmi et al. revealed the regulatory role of ArlRS in autolysis is limited to methicillin-sensitive *S. aureus* (MSSA) strains, with autolysis in MRSA controlled by a distinct, yet undefined mechanism [15]–[17]. We demonstrate the ArlRS system controls *S. aureus* agglutination by negatively regulating the expression of the Giant Staphylococcal Surface Protein (GSSP) [18], also called the extracellular matrix binding protein homologue (Ebh) [19]. We also demonstrate the ArlRS system is essential for pathogenesis in a rabbit model of sepsis and infective endocarditis.

## Results

### *S. aureus* agglutinates in the presence of human plasma

**Figure 1:**
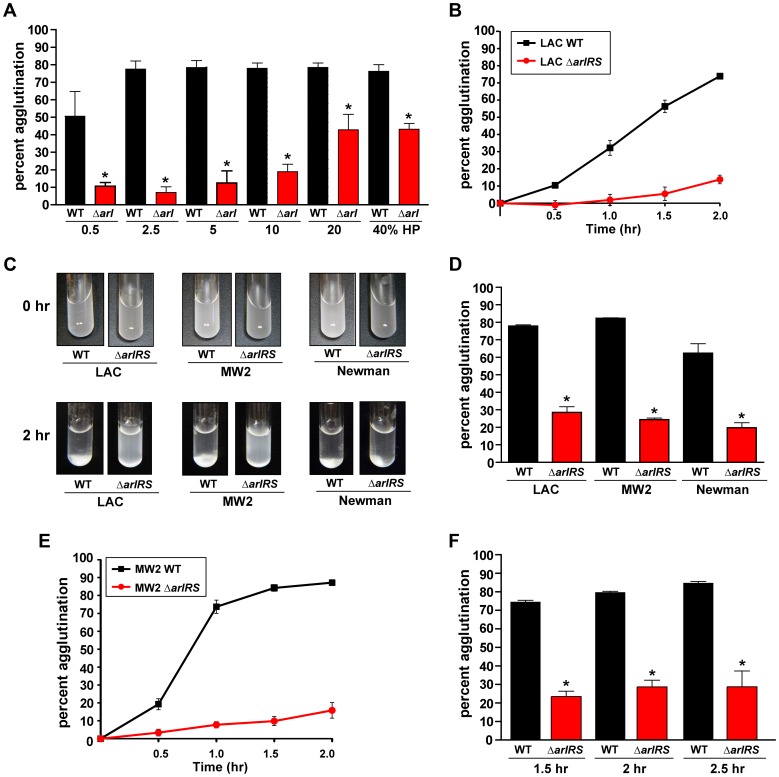
Agglutination of *S. aureus* is induced by human plasma and dependent on ArlRS. Addition of HP to wildtype *S. aureus* strains LAC, MW2, and Newman resuspended in PBS induces rapid clumping of the cells, resulting in a clearing of the supernate due to sedimentation of the clumps. Mutation of *arlRS* inhibits clumping, preventing sedimentation leaving the suspension cloudy. **A**) A dose response of human plasma (HP) ranging from 0.5%–40% was incubated with LAC-WT and LAC Δ*arlRS*. **B**) A time course of LAC-WT and LAC Δ*arlRS* at 2.5% HP over 2 hr. **C**) Visualization of tube agglutination. Top row: Cells resuspended in PBS at time 0 hr after the addition of HP. Bottom row: cells after 2 hr in the presence of HP. **D**) Quantitative measure of the decrease in optical density as wildtype *S. aureus* strains clump while their isogenic *arlRS* mutants do not. **E**) A time course of MW2-WT and MW2 Δ*arlRS* at 2.5% HP over 2 hr. **F**) Agglutination of LAC-WT and LAC Δ*arlRS* with 2.5% citrated HP at 1.5, 2, and 2.5 hr. Bars are standard deviations of at least three replicates, and significant differences (p<0.01) as determined by Student's *t*-test are indicated by “*”.

To understand the agglutination process more clearly and determine how closely these interactions resemble biofilm communities, we developed an approach to quantify *S. aureus* agglutination *in vitro* using human plasma. Briefly, early growth phase *S. aureus* cells were prepared and resuspended in PBS, and the cell suspensions were incubated with an increasing concentration of human plasma (HP) to determine a dose response statically at room temperature (Fig. 1A). Aliquots of supernate were removed over time for turbidity readings to assess the amount of remaining cells (Fig. 1B). We define agglutination as the production of large clumps that clear the supernate up to 70% of the initial cells present (Fig. 1C). After repeated trials, we selected 2.5% HP (1∶1 mixture of HP and heparin/dextran) as the standard amount used in experiments and 2 hr incubation as the standard incubation time (unless otherwise noted). As a control, cell suspensions incubated without HP took over 16 hr for cells to sediment and reach 70% agglutination (data not shown), demonstrating the importance of the plasma proteins to the phenotype.

Several divergent *S. aureus* strains were tested for their ability to agglutinate, including USA300 (LAC-WT), USA400 (MW2), and Newman. The addition of 2.5% HP induced clump formation within 2 hr for all strains tested, which could be visualized as a white mass at the bottoms of the tubes (Fig. 1C). LAC-WT, MW2, and Newman displayed 78%, 82%, and 70%, agglutination, respectively 2 hr post-addition of 2.5% HP (Fig. 1D). A time course of MW2 was performed (Fig. 1E), revealing that this strain agglutinated at a faster rate than LAC-WT (Fig. 1B). The effect of anticoagulant on *S. aureus* agglutination (heparin/dextran sulfate) was also tested, and the cells sedimented at the same rate as those without HP (data not shown). To assess the impact of other anticoagulants, LAC-WT agglutination was tested with 2.5% citrated HP over time and revealed similar results (Fig. 1F), further demonstrating the anticoagulant does not affect *S. aureus* clumping. Thus, we have developed a straightforward and simple assay to quantify *S. aureus* agglutination with HP that shows a robust phenotype conserved across strains.

### Fibrinogen is the major host factor in HP contributing to *S. aureus* agglutination

**Figure 2:**
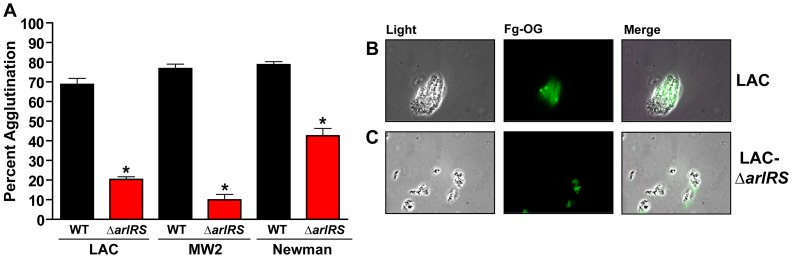
Fibrinogen is the major factor in HP contributing to *S. aureus* agglutination. **A**) 18.5 µg/mL of fibrinogen (Fg) induces agglutination of *S. aureus* wildtype strains similarly to 2.5% HP. Mutants in *arlRS* were unable to agglutinate with Fg. **B/C**) Fg-OG was used to visualize LAC-WT (**B**) clumps and LAC Δ*arlRS* (**C**) clumps using fluorescence microscopy. Light, fluorescent, and merged images are shown, respectively. Bars are standard deviations of at least three replicates, and significant differences (p<0.01) as determined by Student's *t*-test are indicated by “*”.

Previous studies on agglutination indicate that the human matrix protein, fibrinogen (Fg), is a major component of HP required to induce clumping in *S. aureus* [20]. To test the potential role of Fg in agglutination, the protein was added at a final concentration of 18.5 µg/mL (roughly the concentration calculated to be present in 2.5% HP). At this concentration, Fg induced agglutination similar to HP in all *S. aureus* wildtype strains tested with LAC-WT, MW2, and Newman displaying 73%, 82%, and 81% agglutination after 2.5 hr, respectively (Fig. 2A). This agglutination was specific to Fg; as a dose-response of fibronectin added up to 25 times the amount found in 2.5% HP was unable to agglutinate wildtype *S. aureus* strains comparable to HP (data not shown). Similarly, the addition of a dose-response of human serum was insufficient to agglutinate strains to levels equivalent to HP (data not shown).

To visualize the agglutinated clumps, Fg conjugated to the fluorophore Oregon Green (Fg-OG) was used in the agglutination assay, and the clumps were examined using fluorescence microscopy. LAC-WT was used as a representative strain in the experiment. Similar to the tube-based assay, LAC-WT formed large aggregates as seen with light microscopy. In fluorescence mode it was evident that Fg-OG was incorporated and embedded in the clumps (Fig. 2B). Our observations indicate that Fg is the major component of human plasma promoting agglutination and this ECM protein becomes an integral component of the clump.

### *S. aureus* genetic factors essential for agglutination

**Figure 3:**
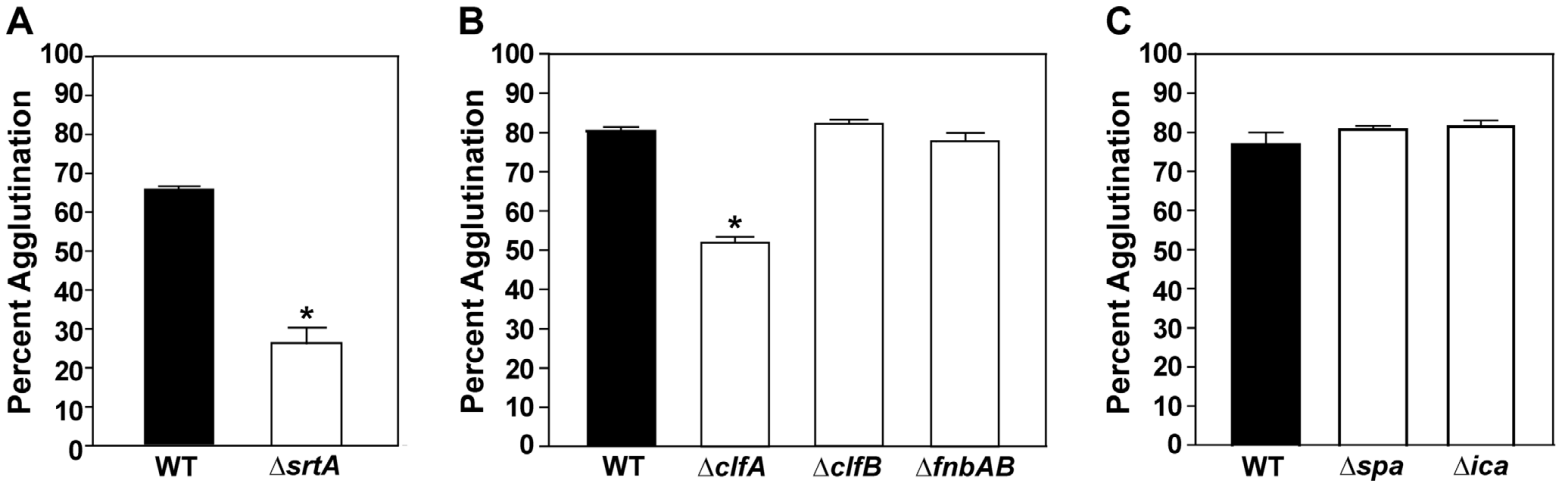
MSCRAMMs are required for agglutination. The tube-based agglutination assay was performed and quantified with LAC-WT and mutant strains. **A**) LAC-WT compared to a sortase mutant (LAC Δ*srtA*; 26% agglutination). **B**) LAC-WT compared to mutations of individual MSCRAMMs (*clfA*, *clfB*, and *fnbAB*) at 1.5 hr post HP addition. The *clfA* mutant displayed 55% agglutination. **C**) LAC-WT compared to mutants in protein A (*spa*) and *ica* (encoding PIA) at 2 hr post HP addition. Bars are standard deviations of at least three replicates, and significant differences (p<0.001) as determined by Student's *t*-test are indicated by “*”.

Several genetic factors encoded by *S. aureus* required for agglutination have been described [21] including clumping factor A (ClfA) [13], [22], a fibrinogen-binding protein and a member of the microbial surfaces components recognizing adhesive matrix molecules (MSCRAMMs), and the two coagulating proteins, coagulase A (Coa) and von Willebrand factor binding protein (vWbp) [14]. These factors, along with several other MSCRAMMs, were shown to be involved in abscess formation during systemic infection and contribute to persistent disease [13], [14], [23]. To compare our agglutination assay to these published reports, we examined the ability of a strain with a mutation in sortase A (Δ*srtA*), a transpeptidase responsible for proper MSCRAMM localization, to agglutinate [24]. The LAC Δ*srtA* mutant displayed a defect in the ability to agglutinate compared to LAC-WT (Fig. 3A). Newman Δ*srtA* was also assessed for the ability to agglutinate. Newman Δ*srtA* was indeed deficient for agglutination at early time points, although not to the same degree LAC Δ*srtA* (data not shown).

Since a number of MSCRAMMS have defined roles in adherence and biofilm formation, we also assessed their role in *S. aureus* agglutination. To characterize the contribution of individual MSCRAMMs, we examined whether strains with mutations in the coding sequences of clumping factor A (ClfA), clumping factor B (ClfB) [25], fibronectin binding proteins (FnbpA and B) [26], or protein A (Spa) [27] could agglutinate in our assay [13]. A LAC *clfA* mutant showed a significant defect at early time points, displaying only 53% agglutination compared to LAC-WT which exhibited 80% agglutination at the same time point (Fig. 3B). Interestingly, the Δ*clfB*, Δ*fnbpAB*, and Δ*spa* mutants, in strain LAC-WT, displayed no phenotype in the agglutination assay (Fig. 3B & 3C). Due to the important role of the polysaccharide intercellular adhesin (PIA) in biofilm and aggregate formation [12], we also tested strain LAC-WT with a deletion of the *ica* genes, which encode the PIA biosynthesis components, and again no defect was observed in agglutination of the LAC Δ*ica* mutant (Fig. 3C). To assess the generality of the observations, the contribution of MSCRAMMs to agglutination in strain Newman was also determined and revealed strikingly similar results (data not shown). Taken together, these studies demonstrate that *in vitro* agglutination of *S. aureus* requires MSCRAMMs, specifically *clfA*, for proper interactions with matrix proteins, and many known factors important for biofilm formation are not essential for the agglutination phenotype.

### ArlRS regulates agglutination

**Figure 4:**
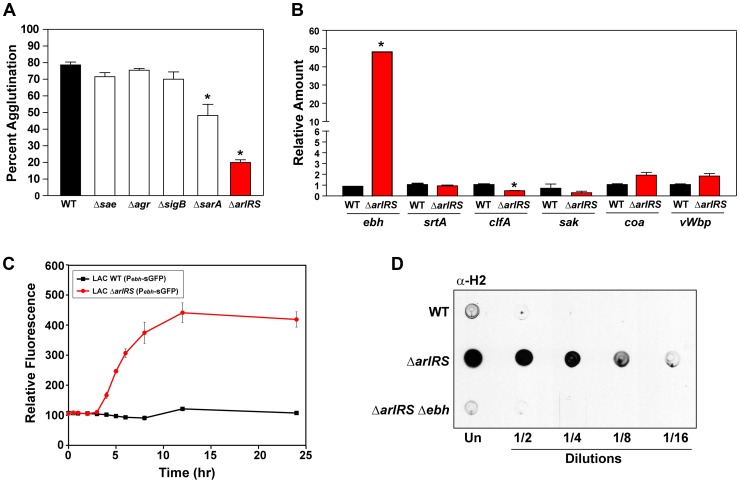
ArlRS contributes to agglutination through the repression of *ebh*. **A**) Tube-based agglutination assays were performed with regulator mutants in LAC-WT and quantified 2 hr post HP addition. The strains included LAC-WT and mutants in *sae*, *agr*, *sigB*, *sarA*, and *arlRS*. **B**) Transcript levels of *ebh*, *srtA*, *clfA*, *sak*, *coa*, and *vWbp* were assessed by qPCR in both LAC-WT (black bars) and LAC Δ*arlRS* (red bars). The level of transcription is normalized to *gyrB* as a control. **C**) A time course of LAC-WT (black squares) and LAC Δ*arlRS* (red circles) with the P*_ebh_*-sGFP reporter. Growth (OD_600_) and fluorescence readings were taken over time and relative fluorescence was plotted. **D**) Dot immunoblot of sheared surface proteins from LAC-WT, LAC Δ*arlRS*, and LAC Δ*arlRS* Δ*ebh* strains that also contained a mutation in *spa* to prevent non-specific antibody binding. Serial dilutions of surface proteins were spotted on a nitrocellulose membrane and probed using antibodies specific for the H2 domain of GSSP. Bars are standard deviations of at least three replicates, and significant differences (p<.001) as determined by Student's *t*-test are indicated by “*”.

**Figure 5:**
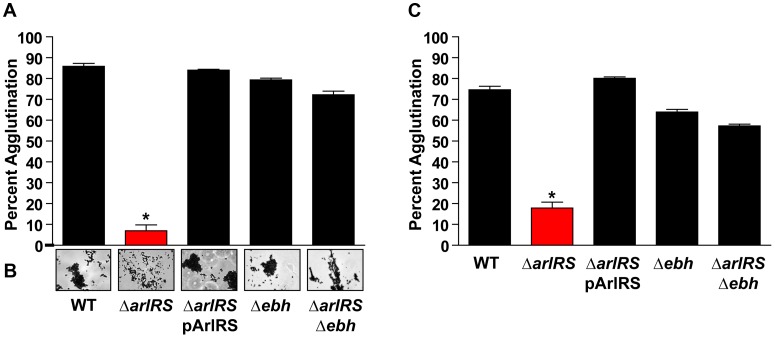
Inactivation of *ebh* restores the agglutination phenotype of an *arlRS* mutant. **A**) LAC-WT, LAC Δ*arlRS*, LAC Δ*arlRS* pArlRS, LAC Δ*ebh*, and LAC Δ*arlRS* Δ*ebh* were incubated with 2.5% HP (**A**) or 18.5 µg/mL Fg (**C**) and assayed for agglutination after 2 hr. **B**) Light microscopy images of clumps 2 hr after HP addition showed all strains formed tight clumps similarly to LAC-WT except LAC Δ*arlRS*, which formed a loose collection of cells. Bars are standard deviations of at least three replicates, and significant differences (p<0.001) as determined by Student's *t*-test are indicated by “*”.

To better understand the mechanisms that control agglutination, we assessed the impact of mutations in global regulators on the ability of *S. aureus* to agglutinate in the presence of HP or Fg. We initially selected regulators with reported roles in biofilm formation, including SarA [28], sigma factor B (SigB) [29], *agr* quorum sensing [30], SaeRS [31], and ArlRS [15], [32]. The addition of HP revealed that strains with mutations in *agr*, *sigB*, or *saeRS* displayed no phenotype in the assay, while a *sarA* mutant had a minor defect in clumping (Fig. 4A). Addition of purified Fg revealed the same result (data not shown). Surprisingly, we observed that a Δ*arlRS* mutant had a dramatic phenotype in agglutination compared to LAC-WT (Fig. 4A). The agglutination inhibition occurred throughout a LAC Δ*arlRS* time course (Fig. 1B). It was also conserved across doses of HP (from 0.5%–40%; Fig. 1A) and in the presence of other anti-coagulants, such as citrated HP (Fig. 1F). Similar results were obtained with MW2-WT and MW2 Δ*arlRS* strains (Fig. 1E and data not shown). This phenotype could be complemented with a plasmid encoding the *arlRS* genes under regulatory control of the native promoter (Fig. 5A), demonstrating that the absence of the *arlRS* genes was responsible for the observed phenotypes. The *arlRS* mutation was reconstructed in the MW2 and Newman backgrounds and assessed for agglutination with either HP (Fig. 1C and 1D) or purified human Fg (Fig. 2A). In all strains tested, agglutination was markedly inhibited when the *arlRS* genes were disrupted.

To better appreciate the agglutination phenotype, Fg-OG was utilized to visualize the clumps formed by LAC-WT and its isogenic *arlRS* mutant. As noted above, LAC-WT bound Fg-OG and formed large, tight clumps with embedded Fg, while the LAC Δ*arlRS* clumps were small, spatially separated, with only trace levels of Fg-OG detectable (Fig. 2B). These observations, using multiple assessments of agglutination, demonstrate that strains missing the ArlRS two-component system prevent agglutination by inhibiting proper interactions with Fg.

### The *ebh* gene is overexpressed in an *arlRS* mutant

With the identification of ArlRS as a regulator of agglutination, we examined a previously published microarray to determine how the two-component system contributes to agglutination [33]. Surprisingly, mRNA transcript abundance of factors known to be involved in agglutination, such as ClfA, sortase and the coagulases, were not identified as being significantly altered in an *arlRS* mutant. To confirm these observations in the LAC-WT strain background, we employed quantitative real time PCR (qPCR) to determine transcript levels of these factors in LAC-WT and LAC Δ*arlRS*. There were no significant differences in transcript levels of sortase and the coagulases in the *arlRS* mutant compared to LAC-WT (Fig. 4B). However, *clfA* transcript expression was reduced two-fold in LAC Δ*arlRS* compared to LAC-WT. The transcript level of staphylokinase (*sak*), which is known to inhibit clumping through plasminogen activation [34], [35], was also compared between LAC-WT and LAC Δ*arlRS* and revealed no significant changes in expression (Fig. 4B). These regulatory observations suggested ArlRS was controlling agglutination by a unique, undefined mechanism.

The reported microarray revealed striking transcriptional changes in a set of genes, *ebhA* and *ebhB*, that had the potential to be involved in agglutination [33]. Characterization of these genes in Mu50 and N315 suggested *ebhA* and *ebhB* were originally a single open reading frame and a frameshift mutation resulted in their permanent separation [19]. Analysis of these genes in other *S. aureus* strains, including MW2 and LAC-WT, revealed these strains contained one intact gene, termed *ebh*. The *ebh* gene is the largest open reading frame on the *S. aureus* chromosome at 33 kb, effectively comprising 1% of the genome [19]. Ebh, also called Giant Staphylococcal Surface Protein (GSSP), is predicted to be membrane anchored and protruding from the cell surface in a fiber-like manner [36], [37]. The protein contains numerous sugar binding and albumin binding repeat domains that are thought to be important for binding extracellular matrix proteins [19]. To test whether *ebh* is upregulated in the LAC Δ*arlRS* mutant, we employed qPCR to assess transcript levels. During early logarithmic growth, *ebh* expression was increased almost 50 fold in LAC Δ*arlRS* compared to LAC-WT (Fig. 4B). To confirm with a different assay, the *ebh* promoter was fused to sGFP to create a transcriptional reporter, and the levels of GFP in LAC Δ*arlRS* increased markedly throughout growth compared to LAC-WT (Fig. 4C).

To address the production of GSSP from the *ebh* gene, the H2 domain of GSSP was purified and antibodies were generated. The H2 domain is a 392-residue peptide composed of three sugar-binding motifs in one of the repeat regions [19]. Dot blots to assess GSSP production were performed in a manner similar as done recently with *S. epidermidis* [18], and mutations in Protein A (Spa) were engineered into the strains to eliminate background response on the blots. As shown in Figure 4D, GSSP levels are low in LAC *spa* mutant and they rise dramatically in a LAC Δ*arlRS spa* double mutant. As a control, a LAC Δ*arlRS* Δ*ebh spa* mutant was constructed and the GSSP response was eliminated, demonstrating the specificity of the H2 antibody. These findings revealed that a functional ArlRS is required to repress the transcription of *ebh*, preventing the expression of GSSP on the surface of the cell.

### ArlRS repression of *ebh* allows agglutination

To assess the potential role GSSP may play in inhibiting agglutination, LAC Δ*ebh* single mutant and LAC Δ*arlRS* Δ*ebh* double mutant strains were constructed in the LAC-WT background. Using the agglutination assay described above, the LAC Δ*ebh* mutant displayed no phenotype in the presence of either HP or Fg (Fig. 5A and C respectively). Importantly, the LAC Δ*arlRS* Δ*ebh* double mutant displayed an agglutination level similar to LAC-WT in either HP or Fg (Fig. 5A and C), effectively eliminating the phenotype observed with LAC Δ*arlRS*. This observation suggests that the overexpression of GSSP in an *arlRS* mutant is responsible for the inability of LAC Δ*arlRS* to agglutinate, and this phenotype is abrogated upon mutational disruption of GSSP expression in an *arlRS* mutant background. To visualize agglutination by each strain, light microscopy was performed. The images revealed that the LAC Δ*ebh* and LAC Δ*arlRS* Δ*ebh* mutants both formed tight clumps similar to LAC-WT (Fig. 5B), while the LAC Δ*arlRS* mutant cells associated in loose collections, consistent with our previous observations. Together these data indicate the overexpression of the *ebh* gene in the LAC Δ*arlRS* mutant is the major factor responsible for the inhibition of agglutination.

Another large surface protein, SasC, is up-regulated more than 10-fold in an *arlR* mutant [33], and we investigated the potential that SasC plays a similar function as GSSP in agglutination. For this experiment, we constructed a LAC Δ*arlRS* Δ*sasC* double mutant using a *sasC*::φΣ insertion from the Nebraska Transposon Mutant Library [38]. However, LAC Δ*arlRS* Δ*sasC* strain showed the same agglutination phenotype as the LAC Δ*arlRS* mutant (data not shown), suggesting that, unlike GSSP, SasC is not involved in blocking agglutination under these conditions. The *sasC* mutation was also introduced into the LAC Δ*arlRS* Δ*ebh* strain, and again this additional mutation did not alter agglutination phenotypes (data not shown).

### Incorporation of fibrinogen into clumps influences size

**Figure 6:**
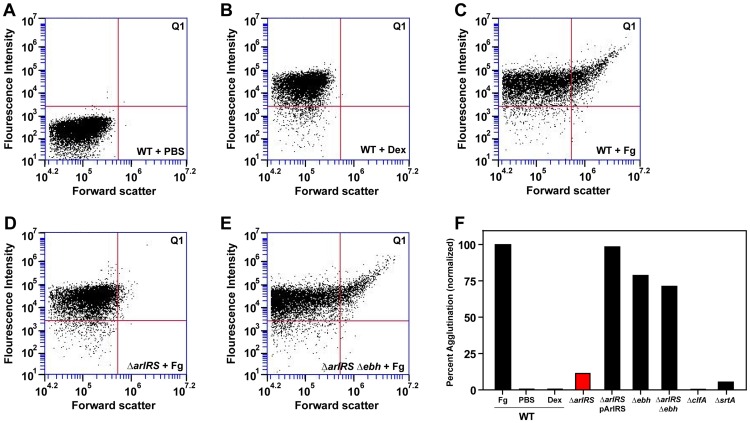
*S. aureus* agglutinates through the incorporation of Fg. All strains were grown in BHI, washed in PBS, incubated with controls or Fg-OG, and analyzed by flow. A shift in population into upper-right quadrant Q1 was considered positive for Fg-OG incorporation and agglutination. **A**) LAC-WT in PBS as a control. **B**) LAC-WT incubated with dextran sulfate conjugated to OG as a control. The following strains were incubated with Fg-OG and assayed by flow: LAC-WT (**C**), LAC Δ*arlRS* (**D**), and LAC Δ*arlRS* Δ*ebh* (**E**). Strains LAC Δ*arlRS* pArlRS and LAC Δ*ebh* were also assayed by flow with Fg-OG and agglutinated similarly to LAC-WT. **F**) Plot of flow agglutination results with all strains relative to LAC-WT (set to 100%). As controls, single mutants in *clfA* and *srtA*, were also included in the flow assay.

To define the roles of ArlRS and GSSP in the agglutination phenotype, flow cytometry was employed to assess agglutination. Background fluorescence controls (LAC-WT and LAC-WT incubated with dextran sulfate conjugated to Oregon Green) were set as a baseline to less than 0.1% (Fig. 6A and B). A shift in population into the upper right quadrant (Q1) was positive for Fg-OG incorporation and agglutination. In Figure 6, representative examples of the flow observations are shown (Fig. 6C–E), along with data from all of the strains normalized to LAC-WT set to 100% (Fig. 6F). Incubating LAC-WT with Fg-OG revealed a shift of 12.1% of the population from the left quadrants into Q1, indicating LAC-WT bound Fg-OG in a productive manner, resulting in clump formation (Fig. 6C). Similar to our previous observations, LAC Δ*arlRS* did not agglutinate and displayed less than 2% shift into Q1 (Fig. 6D). However, the shift does indicate the Δ*arlRS* mutant can bind Fg to some degree. The removal of GSSP in the LAC Δ*arlRS* Δ*ebh* double mutant resulted in an 8.5% shift in population into Q1, translating to 70% agglutination relative to LAC-WT (Fig. 6E and F). Again, the introduction of the Δ*ebh* mutation restored much of the LAC Δ*arlRS* defect. The LAC Δ*arlRS* complemented strain, and the LAC Δ*ebh* mutant, behaved similar to LAC-WT, agglutinating 98% and 78% relative to LAC-WT, respectively (Fig. 6F). As controls, single mutants in *clfA* and *srtA*, were also assessed in the flow cytometry assay. LAC Δ*clfA* and LAC Δ*srtA* agglutinated to 0% and 5% of LAC-WT respectively (Fig. 6F), demonstrating the importance of ClfA to agglutination. These strains appear to exhibit a more severe phenotype in the flow cytometry assay compared to the gravity agglutination assay. However, the flow cytometry assay is only assessing Fg binding and incorporation, and the results shown are gated (Fig. 6F), while experiments using HP contained many other host ECM proteins that potentially additively contribute to agglutination. These flow data corroborate our other agglutination observations and confirm the role of GSSP in an *arlRS* mutant as a major inhibitor of agglutination.

### SEM images of clumps

**Figure 7:**
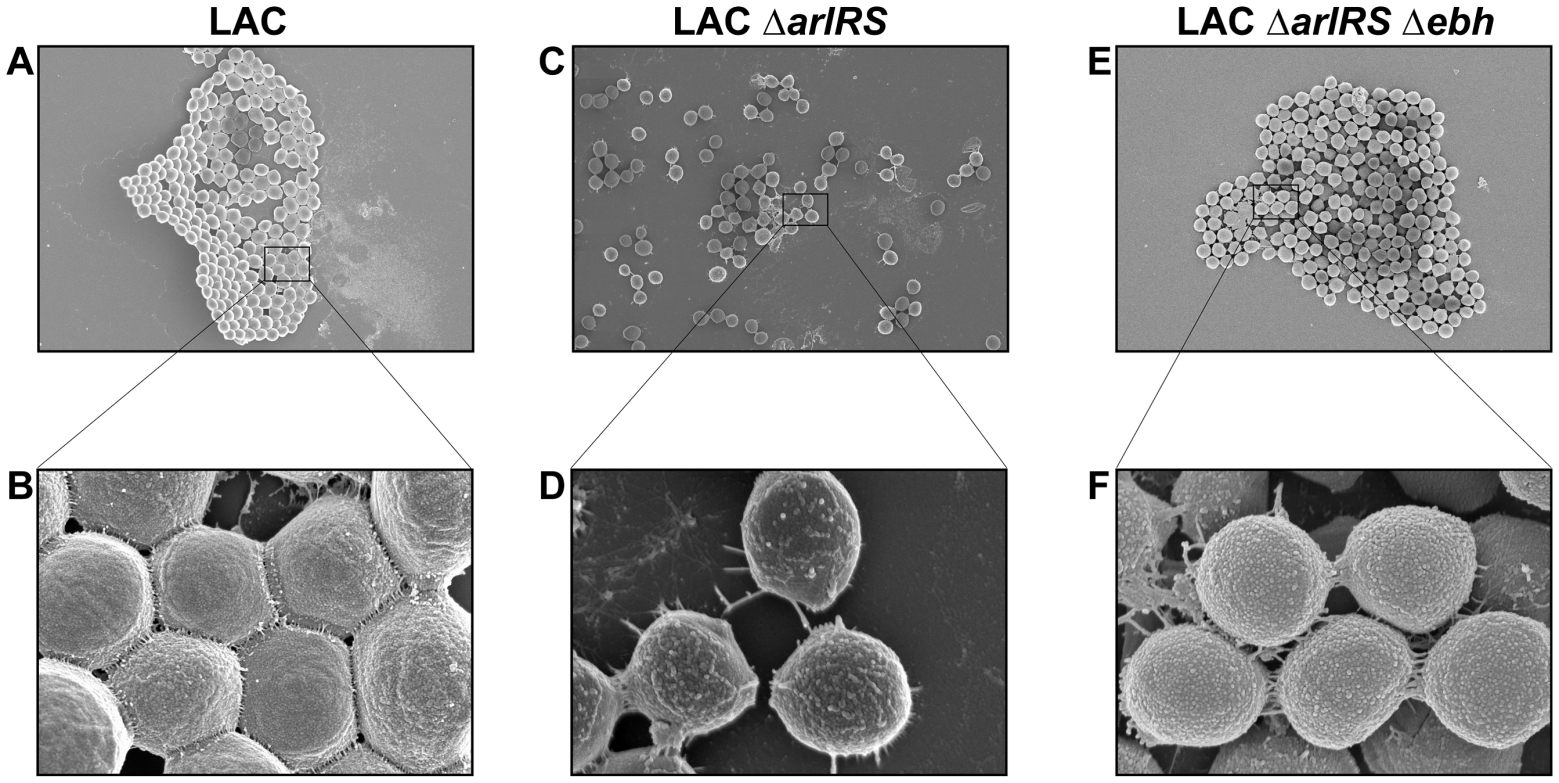
SEM of LAC-WT, LAC *ΔarlRS*, and LAC *ΔarlRS Δebh* clumps. SEM images of LAC strains agglutinated with Fg and fixed on coverslips. Images were taken on a Hitachi S-4800 scanning electron microscope. Top row (**A**, **C** and **E**) shows representative images taken at a magnification of 4.5K and 10 µm. Bottom row (**B**, **D** and **F**) shows magnification at 45K and 1 µm. The strains were as follows: LAC-WT (**A**, **B**); LAC Δ*arlRS* (**C**, **D**); and LAC Δ*arlRS* Δ*ebh* (**E**, **F**). Agglutinated clumps and SEM images of LAC Δ*ebh* and LAC Δ*arlRS* pArlRS displayed similar characteristics to LAC WT (data not shown).

To further assess whether the LAC-WT and LAC Δ*arlRS* clumps are morphologically distinct, scanning electron microscopy (SEM) was employed. Images revealed LAC-WT formed large, tight clumps in the presence of Fg (Fig. 7A) with visible cross-links binding the cells together at a magnification of 45K (Fig. 7B). Clumps formed by LAC Δ*arlRS* lacked the structured cross-links displayed by LAC-WT at high magnification, and instead loose collections with long string-like fibers appeared to connect the cells (Fig. 7C and D), resulting in abnormal clump formation. Agglutination of LAC Δ*ebh*, LAC Δ*arlRS* complemented, and LAC Δ*arlRS* Δ*ebh* displayed similar characteristics to LAC-WT, revealing tight interactions between cells (data not shown and Fig. 7E and F, respectively). The flow data suggests that the LAC Δ*arlRS* can bind some level of Fg, however, the clumps are characteristically distinct from LAC-WT in SEM, suggesting the *arlRS* mutants interact with Fg inefficiently. Importantly, the deletion of *ebh* in an *arlRS* background rescues agglutination, providing further evidence that the proper regulation of GSSP is essential.

### Constitutive expression of *ebh* by the *fabI* promoter prevents agglutination

**Figure 8:**
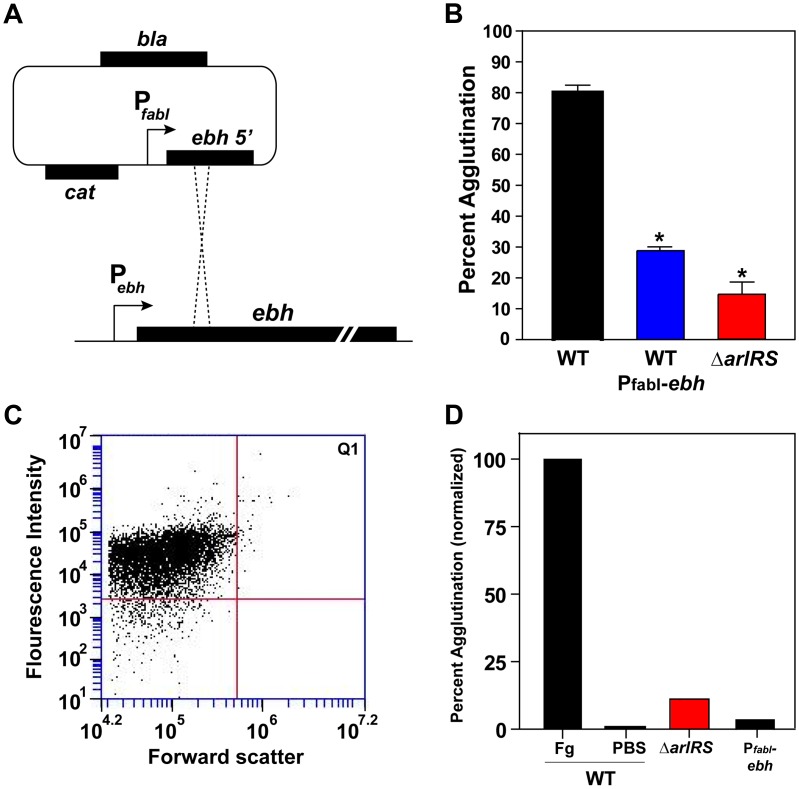
Constitutive expression of *ebh* prevents agglutination in LAC-WT. **A**) Schematic of the chromosomal integration of the *fabI* promoter upstream of the *ebh* gene, displacing the *ebh* promoter, on the LAC-WT chromosome. The construct results in the constitutive expression of the *ebh* gene. **B**) LAC-WT, LAC P*_fabI_*-*ebh*, and LAC Δ*arlRS* were tested and compared in the tube-based agglutination assay. **C**) LAC P*_fabI_*-*ebh* strain was assessed for agglutination using the flow assay, similar as outlined in Figure 6. **D**) The results of the flow assay with LAC-WT, LAC P*_fabI_*-*ebh*, and LACΔ*arlRS* were compared after 2 hr of agglutination. LAC-WT agglutination with Fg-OG was set to 100%, and a PBS negative control was included. Bars are standard deviations of at least three replicates, and significant differences (p<0.001) as determined by Student's *t*-test are indicated by “*”.

To confirm that GSSP is the major factor responsible for the inhibition of agglutination in an *arlRS* mutant, we replaced the wild-type *ebh* promoter with the *fabI* promoter in LAC-WT. P*_fabI_* was inserted into the chromosome of LAC-WT displacing the *ebh* promoter, resulting in a strain that constitutively drives a low level of *ebh* expression (Fig. 8A). LAC P*_fabI_*-*ebh* was assessed in the agglutination assay, where it displayed a defect in clumping similar to LAC Δ*arlRS* (Fig. 8B). Further, this construct was tested with the flow assay, and similar to LAC Δ*arlRS*, the LAC P*_fabI_*-*ebh* strain displayed a population shift of less than 1% into Q1, indicating little Fg-OG incorporation and clumping (Fig. 8C). Using the flow assay, both LAC Δ*arlRS* and LAC P*_fabI_*-*ebh* agglutinated less than 10% relative to LAC-WT (Fig. 8D). These findings confirm that increased expression of *ebh* is responsible for the inhibition of agglutination in an *arlRS* mutant.

### ArlRS is attenuated in pathogenesis

With the newly identified role of ArlRS in agglutination, we evaluated Δ*arlRS* mutants using *in vitro* and *in vivo* assessments of pathogenesis. Preliminary reports suggest that ArlRS regulates alpha toxin (Hla) [17], an important virulence factor in animal models [39], [40], but this gene was not identified as significantly regulated in the published microarray [33]. To determine whether ArlRS plays a role in the transcriptional regulation of alpha toxin, *hla* transcript levels were measured by qPCR in both LAC-WT and LAC Δ*arlRS*. Transcript levels of *hla* displayed no significant differences between the strains (data not shown). Since Hla is also regulated at the post-transcriptional level [41], rabbit red blood cell lysis titers were performed, and again no significant differences were observed between LAC-WT and LAC Δ*arlRS* (data not shown). These observations indicate that the ArlRS system does not regulate Hla in CA-MRSA USA300 strains or potentially other clinical isolates.

The ArlRS system was identified as being important for pathogenesis in murine models of systemic infection in a random mutagenesis screen to discover virulence determinants [42]. However, these experiments were performed using laboratory strains and the phenotypes have not been reassessed. In this study, we took advantage of rabbit models, which more accurately replicates the disease state seen in humans than mouse models [43]. Importantly, agglutination by other pathogens has been shown to be required for virulence in this model [44]. With the newly identified role of ArlRS in *S. aureus* agglutination, and the established link between agglutination and pathogenesis [13], [14], [44], we tested the contribution of ArlRS to pathogenesis using a combined model of sepsis and infective endocarditis in rabbits. The high level of Hla production in USA300 strains is lethal to rabbits, making endocarditis vegetation formation difficult to monitor [45]. To circumvent these difficulties we used the relevant USA400 MW2 isolate for these experiments. A dose of ∼10^6^–10^7^ CFU MW2-WT or MW2 Δ*arlRS* was injected into rabbits via the marginal ear vein. Rabbits were examined four times a day over a four-day period, and were euthanized upon signs of illness or at the completion of the experiment. Hearts were removed and assessed for the presence of vegetations. Vegetations at the valve sites were removed, weighed, homogenized, and plated for bacterial counts.

**Figure 9:**
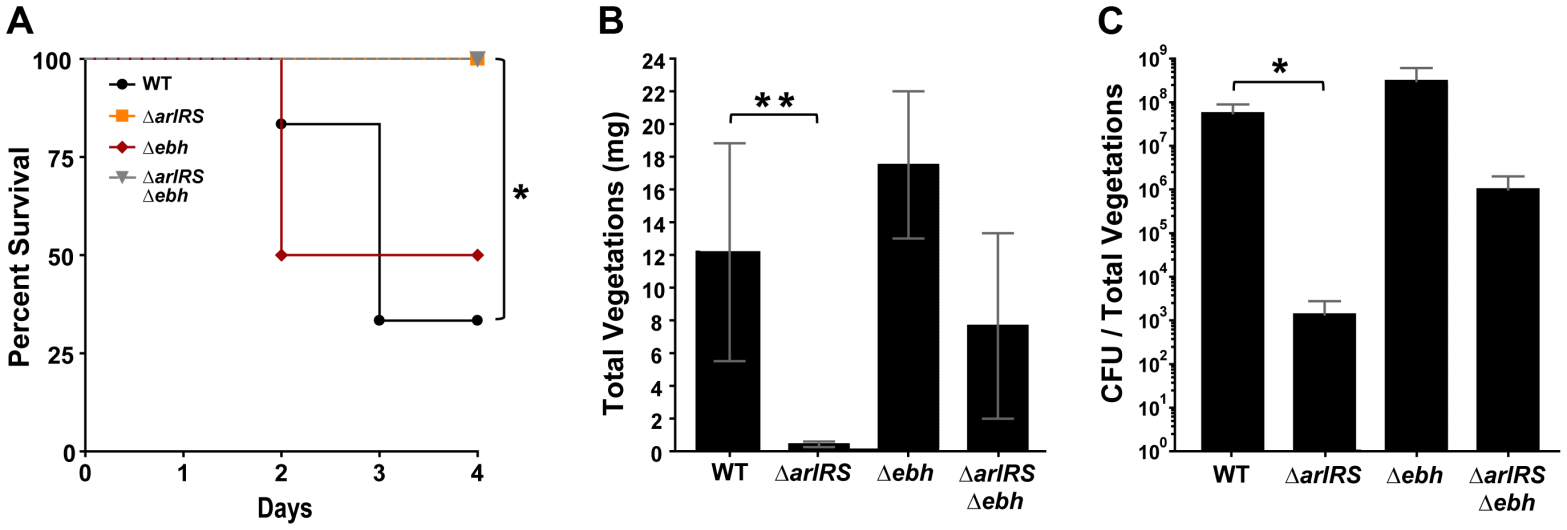
Strains lacking ArlRS are defective in a rabbit model of sepsis and endocarditis. NZW rabbits (either sex) were anesthetized and their aortic valves damaged with hard plastic catheters for 2 hr. The catheters were removed, and their necks sutured closed. MW2-WT and mutant strains were injected intravenously; animals were monitored for health status for up to 4 days. Percent survival (**A**), vegetation weights (**B**), and log CFUs/total vegetations per animal (**C**) were recorded at the time of death or upon completion of the experiment at day 4. Bars on log CFUs indicate standard deviations. *p = 0.01 (percent survival), **p = 0.005 (vegetation size) and *p = 0.03 (CFU/total vegetations). Statistical significance was determined using a two-tailed, Mann Whitney (non-parametric) test or Log-rank (Mantel-Cox) Test for survival.

Four of the six rabbits infected with MW2-WT died before day four: one rabbit on day two; and three rabbits on day three (Fig. 9A). All rabbits infected with MW2-WT developed vegetations with an average weight of 12 mg and 2.6×10^7^ CFU/vegetations (Fig. 9B and C). In contrast, all rabbits infected with MW2 Δ*arlRS* survived until day four (Fig. 9A) and had either no vegetations or very small vegetations, which was statistically significant (p<0.05). The average vegetation weight derived from these infections was less than 0.5 mg (Fig. 9B) and bacteria were recovered from only one vegetation, which contained less than 1.0×10^4^ CFU (Fig. 9C). All other vegetations recovered from rabbits infected with MW2 Δ*arlRS* were sterile.

To investigate the infective endocarditis defect in the MW2 Δ*arlRS* mutant, a bacterial attachment assay was performed with the MW2-WT and MW2 Δ*arlRS* strains. The sepsis and endocarditis model was performed in a similar manner, except that rabbits were sacrificed 2 hrs post-infection. The hearts were harvested and washed to remove any unbound bacteria, and the bacterial load in the damaged areas was an average of 386 CFU for MW2-WT and 47 CFU for MW2Δ*arlRS*. Although the MW2-WT trended higher, hese differences did not reach statistical significance, suggesting that the ability of MW2-WT or MW2 Δ*arlRS* to adhere to heart valves is similar. These data indicate the reduced virulence of the MW2 Δ*arlRS* mutant in infective endocarditis is not due to a defect in initial attachment to the damaged heart valve.

To determine the impact of GSSP on agglutination *in vivo*, we tested the MW2 Δ*ebh* single and MW2 Δ*arlRS* Δ*ebh* double mutants for the ability to cause infective endocarditis. One of the rabbits challenged with MW2 Δ*ebh* succumbed at day two of the experiment, while all rabbits that were infected with MW2 Δ*arlRS* Δ*ebh* survived the entire experiment (Fig. 9A). The average vegetation weight from rabbits infected with MW2 Δ*ebh* was 17.5 mg and ∼3.1×10^8^ CFU recovered, which are numbers that even exceed MW2-WT (Fig. 9B and C). In comparison, the MW2 Δ*arlRS* Δ*ebh* double mutant developed vegetations with an average weight of 7.7 mg and 1.0×10^6^ CFU recovered (Fig. 9B and C). These are larger than the MW2 Δ*arlRS* vegetations, but the results did not reach statistical significance. Taken together, the ArlRS system is essential for pathogenesis in a rabbit model of sepsis and infective endocarditis, and this phenotype is in part explained by the overexpression of GSSP.

## Discussion

*Staphylococcus aureus* has the ability to form different types of community structures, such as biofilms, that enable this pathogen to persist during infection. It has been well-established that *S. aureus* produces numerous self-adhering factors necessary for establishing these communities, and recent studies have highlighted the importance of matrix protein coated surfaces in biofilm development [46]–[48]. In these *in vitro* models of biofilm formation, it is thought that biofilm initiation occurs though the initial attachment of the cells to the underlying matrix coating, followed by direct cell-cell accumulation. These methods for examining biofilm formation are being recognized as more clinically relevant since they more closely mimic *in vivo* conditions where *S. aureus* attaches to conditioned surfaces [5], [8].

In the absence of a traditional substratum, *S. aureus* has the ability to complex with itself to form aggregates [12], or it can clump in the presence of host proteins [20], [49] through a process called agglutination [13]. The mechanisms of agglutination are complex and only just becoming fully appreciated. When *S. aureus* binds host ECM proteins, the bacterial cells are able to form large clumps, presumably providing protection against various host and antimicrobial exposures. Importantly, in many *in vivo* situations, such as in the bloodstream or peritoneal cavity [50], the local concentration and accessibility of matrix proteins may render agglutination advantageous since direct adherence to an endothelial or epithelial layer may be difficult. Agglutination may also increase *S. aureus* survival *in vivo* by creating microenvironments that retain a functional *agr* quorum-sensing system, thereby facilitating virulence factor expression [49]. Recently, agglutination was linked to the development of abscess communities [13], [21], [51], suggesting that the general ability of *S. aureus* to form tightly packed groups of cells aids survival in various host situations. While there are substantial reports to indicate these communities are important during infection [13], [50], [52], the mechanisms behind the agglutination process remain incompletely defined.

**Figure 10:**
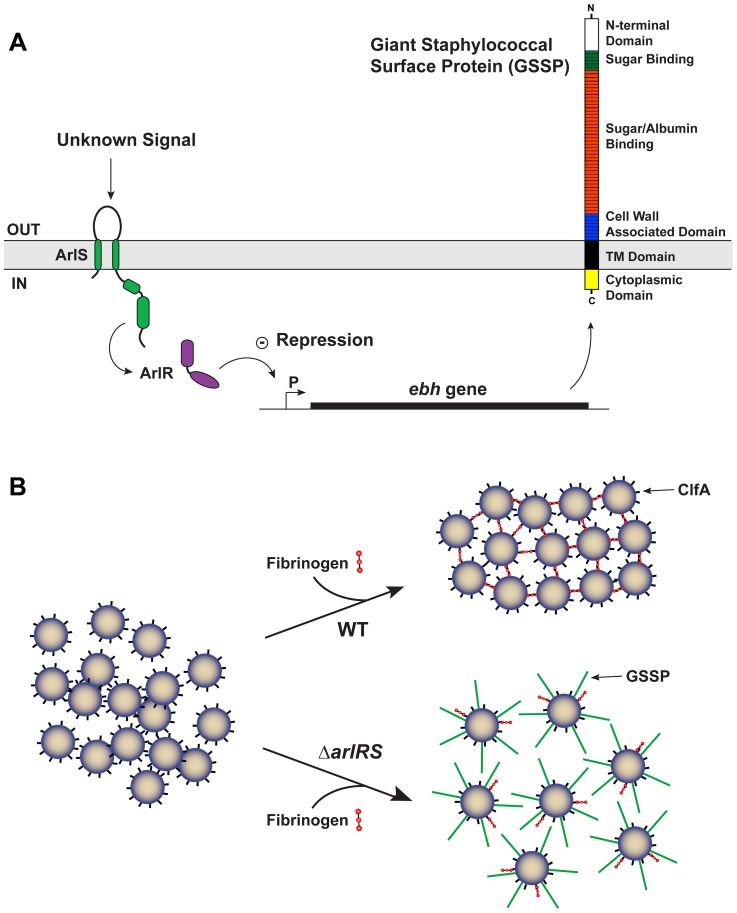
Model of ArlRS and GSSP interconnections and agglutination. **A**) Schematic of the interconnections between the ArlRS two-component system, *ebh* gene, and GSSP. Through an undetermined mechanism, the ArlRS system negatively regulates *ebh*. When the *arlRS* genes are inactivated, *ebh* is overexpressed leading to excess levels of GSSP on the *S. aureus* surface. **B**) Schematic of whole *S. aureus* cells interacting with fibrinogen. With wild-type (WT) cells, they form tight clumps in the presence of fibrinogen due to surface proteins, such as ClfA. The Δ*arlRS* mutants overexpress the *ebh* gene, resulting in extracellular display of GSSP. Through a mechanism that is not known, the full-length GSSP prevents the cells from clumping in the presence of fibrinogen.

In this study, we characterized a new role for the ArlRS two-component system in *S. aureus* agglutination and pathogenesis. Our findings herein demonstrate that ArlRS regulates agglutination by modulating the levels of GSSP (see model in Fig. 10). The *ebh* gene encodes the surface bound protein GSSP, by far the largest protein encoded by the *S. aureus* genome at 1.1 MDa. GSSP is thought to bind matrix components and protect the cell against osmotic pressure changes [19], [53]. Despite these initial reports, remarkably little is known about the physiological and pathogenic role of this giant surface structure. Only one of the repeat domains has been tested for function, called the H2 domain, which was found to bind fibronectin [19]. The *ebh* gene is transcribed at low levels during growth of wildtype strains, but our findings demonstrate that expression and protein levels rise dramatically in the absence of ArlRS (Fig. 4). It is not known whether the response regulator directly binds the *ebh* promoter element (Fig. 10A), as the ArlR binding site has not yet been identified. Therefore the ArlRS repressive effect on GSSP could be direct or indirect through an intermediary. Our findings demonstrate that GSSP inhibits Fg-mediated agglutination, but the mechanism(s) of inhibition is not known (Fig. 10B). One possibility is that GSSP functions like an Fg sink, absorbing excess Fg and preventing proper cell-cell interactions mediated by this matrix protein. However, the published report that GSSP binds fibronectin and not Fg, at least in one repeat domain [19], does not favor this possibility. Another potential inhibitory mechanism is steric hindrance due to the tremendous size and protruding rod-like structure of GSSP [37]. By literally pushing away neighboring cells, clump formation could be prevented, but as of yet, there is no experimental evidence to support this model. Further investigation is necessary to understand the specifics of the mechanism through which GSSP prevents agglutination.

With the growing reported links between agglutination and *S. aureus* pathogenesis [13], [14], we assessed the *in vivo* contribution of the ArlRS system for virulence using a rabbit model of infective endocarditis and sepsis. The endocarditis development program requires *S. aureus* cell-cell interactions and incorporation of matrix proteins to form the vegetation [54], which has parallels to the agglutination mechanism. Our data indicate that wildtype strain MW2, an excellent cause of infective endocarditis [45], resulted in significantly larger cardiac vegetations and higher bacterial load in the vegetations than a strain lacking the ArlRS system. Additionally, more animals infected with MW2-WT succumbed prematurely compared to those infected with the *arlRS* mutant. The mutant defect is likely in the vegetation development pathway, as the adherence of WT and *arlRS* mutant strains to heart valves was similar. An assessment of MW2 Δ*ebh* and MW2 Δ*arlRS* Δ*ebh* mutants indicates that GSSP is a contributor to these rabbit model phenotypes. The MW2 Δ*ebh* was similar, if not superior, in virulence characteristics compared to MW2-WT, while the MW2 Δ*arlRS* Δ*ebh* displayed increased pathogenesis compared to the MW2 Δ*arlRS* mutant. These findings demonstrate that restoring the ability of an *arlRS* mutant to agglutinate also restores pathogenicity, which is consistent with our *in vitro* observations. However, the inability of the MW2 Δ*arlRS* Δ*ebh* strain to completely recover virulence indicates that the GSSP overproduction is not the only ArlRS-regulated factor involved in pathogenesis. Future studies will be needed to further elucidate the ArlRS function *in vivo* during infection.

Our findings are consistent with the hypothesis that bacterial pathogens causing infective endocarditis depend on the ability to agglutinate. In a recent study by Schlievert *et al.*, it was shown that self-aggregation of *Enterococcus faecalis* through its surface aggregation substance (AS), or enhanced aggregation through the combined effects of AS and IgG antibodies against AS, led to increasingly serious diseases compared to organisms lacking AS [44]. In their studies, organisms with AS were better able to cause infective endocarditis than those lacking AS, and the presence of AS IgG antibodies increased the severity of illness. These studies highlight the importance of agglutination in disease causation, and collectively, the data presented herein are consistent with these prior findings, suggesting *S. aureus* agglutination plays a key role in its ability to cause infective endocarditis and sepsis.

The important contribution of ArlRS to the formation of endocarditis vegetations raises questions about the regulation of GSSP in pathogenesis and expression among *S. aureus* strains. The development and resolution of agglutination-based infections could benefit from a control switch like GSSP that modulates agglutination behavior. What environmental or host cues are sensed by the ArlRS system, and how these signals are transmitted to regulate GSSP levels, are all unknown at this time. However, it is tempting to speculate that GSSP levels might be low in the formation of a staphylococcal abscess community and high during the resolution event to break up the community [51]. Similarly, the formation of an endocarditis vegetation could benefit from low GSSP levels to promote *S. aureus* - matrix protein interactions and clump formation. We observed that *ebh* mutants accumulated even larger vegetations than wild-type (Fig. 9B), supporting the hypothesis that the absence of GSSP is beneficial for infective endocarditis. Interestingly, the most frequent causes of infective endocarditis are clonal complex 30 (CC30) strains, causing up to 20% of infections [55]. Bioinformatics analysis indicates the *ebh* gene is truncated in a number of these genomes, including MN8 (data not shown). MN8 is an excellent cause of infective endocarditis [45], and the absence of full-length GSSP further suggests this protein is not important for causing infective endocarditis and is perhaps even inhibitory. However, whether there is a statistically significant trend in *ebh* gene defects across clinical CC30 endocarditis isolates remains to be determined. Looking at other *S. aureus* strain lineages, there is considerable sequence variation in GSSP [56], which could be another contributing factor to agglutination.

In this report, we identified a new function for the *S. aureus* ArlRS system as a regulator of agglutination and pathogenesis. The unexpected discovery that GSSP is the primary ArlRS output controlling agglutination suggests that *S. aureus* has the ability to control clump formation using a surface structure. One goal of our future studies is to decipher the mechanism(s) through which GSSP carries out the agglutination inhibitory activity. The increasing link between *in vivo* agglutination and *S. aureus* pathogenesis emphasize the importance of understanding the regulatory pathways and components involved in clump formation.

## Materials and Methods

### Ethics statement

The animal studies were reviewed and protocol approved by the University of Iowa Institutional Animal Care and Use Committee. The approved protocol was assigned number 1106138. Accordingly, animals were administered pain relieving medications throughout experimentation. Additionally, animals that could not simultaneously maintain upright positions and exhibit normal escape behavior were prematurely euthanized; these criteria are 100% predictive of death in the model used. The University of Iowa is AAALAC accredited, and the centralized facilities meet and adhere to the standards in the “Guide and Care of Laboratory Animals.”

### Reagents and growth conditions

The wildtype and mutant *S. aureus* strains used in this study are listed in Table 1. Trypticase soy broth (TSB) was used to maintain *S. aureus* cultures or prepare overnight cultures for experiments. *Escherichia coli* strains were maintained in Luria-Bertani (LB) broth or on LB agar plates. For the agglutination experiments, all *S. aureus* strains were subcultured and grown in brain heart infusion (BHI) broth. To maintain *S. aureus* mutants and their complementing clones, antibiotics obtained from Sigma-Aldrich were added to the media at the following concentrations: chloramphenicol (Cam) 10 µg/mL, kanamycin 50 µg/mL, erythromycin 10 µg/mL, and spectinomycin 100 µg/mL. To maintain pJMB insertion plasmids, Cam was increased to 30 µg/mL in the media. *E. coli* strains with plasmids were maintained on media supplemented with ampicillin at 150 µg/mL. Other chemical reagents were purchased from Sigma-Aldrich or Fisher Scientific unless otherwise noted.

**Table 1:** Strains and plasmids.

Strain or Plasmid	Description or function	Source or reference
Strains		
*E. coli* strains		
DH5α	Cloning strain	Protein Express
ER2566	Overexpression strain	NEB
*S. aureus* strains		
RN4220	Restriction deficient	[64]
AH1263	USA300 CA-MRSA Erm^S^ (LAC*)	[65]
AH1292	LAC* Δ*agr*::TetM	[31]
AH1558	LAC* Δ*sae*::Spec	[66]
AH1975	LAC* Δ*arlRS*	This work
AH2266	LAC* Δ*arlRS*::Spec	Jovanka Voyich
AH2057	LAC* Δ*ebh*::Kan	This work
AH2633	LAC* Δ*arlRS*::Spec Δ*ebh*::Kan	This work
AH2254	LAC* Δ*srtA*	This work
AH2829	LAC* *clfA*::φNΣ	This work
AH2764	LAC* *ebh*::P*_fabI_*-*ebh*	This work
AH2255	LAC* Δ*clfB::tet*	This work
AH2156	LAC Δ*fnbAB*	Tim Foster
AH2157	LAC Δ*spa*	Tim Foster
AH1712	LAC* Δ*ica::Tet*	This work
AH3007	LAC* *spa*::φNΣ	This work
AH3008	LAC* *spa*::φNΣ Δ*arlRS*::Spec	This work
AH3019	LAC* *spa*::φNΣ Δ*arlRS*::Spec Δ*ebh*::Kan	This work
AH3142	LAC* Δ*arlRS*::Spec *sasC*::φNΣ	This work
AH3143	LAC* Δ*arlRS*::Spec Δ*ebh*::Kan *sasC*::φNΣ	This work
MW2	USA400 CA-MRSA	[67]
AH2453	MW2 Δ*arlRS*::Spec	This work
AH3028	MW2 Δ*arlRS*::Spec Δ*ebh*::Tet	This work
Newman	MSSA	[68]
AH2268	Newman Δ*arlRS*::Spec	This work
AH2580	Newman Δ*clfA*::Erm	Tim Foster
Plasmids		
pCM11	*S. aureus* sGFP expression plasmid, Erm^R^	[61]
pCM11-ebh	P*_ebh_*-sGFP fusion, Erm^R^	This work
pCM28	*S. aureus – E. coli* shuttle vector, Cam^R^	[65]
pArl (pJMB219)	*arlRS* complmenting clone, Cam^R^	This work
pJB38	Mutation generation vector, Cam^R^	[60]
pJMB202	*arlRS* knockout vector, Cam^R^	This work
pJB38-ebh	*ebh*::Kan knockout vector, Cam^R^	This work
pUC19	*E. coli* cloning vector, Amp^R^	NEB
pJMB402	pUC19/P*_fabI_*-*ebh*	This work
pET28-H2	pET28/*ebh* H2-His peptide, Kan^R^	This work

### Recombinant DNA and genetic techniques

*E. coli* DH5α was used as a cloning host for plasmid constructions. Restriction enzymes, DNA ligase, and Phusion DNA polymerase were purchased from New England Biolabs. The plasmid mini-prep and gel extraction kits were purchased from Qiagen. Lysostaphin, used for *S. aureus* DNA extractions, was purchased from Ambion products. Plasmids were electroporated into RN4220 as described previously [57]. Bacteriophage transductions between *S. aureus* strains were performed with phage 80α or 11 as described previously [58]. All oligonucleotides were ordered from IDT Technologies (Coralville, IA) and are listed in Supplementary Table S1. DNA sequencing was performed at the University of Iowa DNA Core Facility or Genewiz, South Plainfield, NJ.

### Agglutination assay

To assess the ability of *S. aureus* to agglutinate, we modified an aggregation assay originally described by Walter et. al. [59] to include the addition of human plasma and other extra cellular matrix proteins. *S. aureus* strains were grown overnight, subcultured in BHI to a starting optical density (OD) at 600 nm wavelength of 0.05, and grown to a final OD_600_ of 1.5. Cells were washed two times with one volume of 1X phosphate buffered saline (PBS) and resuspended in one volume of 1X PBS. Cells were vortexed two times for 20 sec each to ensure all clumps were dispersed. Human plasma (HP) was prepared from donors at the University of Iowa Inflammation Program with all necessary approvals or purchased from Sigma-Aldrich. HP prepared from donors at the University of Iowa was diluted 1∶1 with heparin/dextran sulfate to prevent clotting, and for the purposes of this study, this level of HP was considered a final concentration of 100%. Thus, a 2.5% HP listed throughout is actually 1.25% HP mixed with 1.25% heparin/dextran (vol/vol). Citrated HP purchased from Sigma-Aldrich was resuspended according to manufacturer's instructions and was considered to be a final concentration of 100%. A dose response of HP was used to determine the minimum amount of HP required to induce agglutination, which was 2.5% vol/vol ratio of HP in PBS. For all subsequent experiments HP or Fg was added to initiate agglutination at a final concentration of 2.5% or 18.5 µg/mL (the predicted concentration in 2.5% HP), respectively. As agglutination proceeds large clumps form and quickly fall to the bottom of the tube, leaving the supernate clear of bacterial cells. To quantify agglutination, 100 µL of supernate was removed and measured at an OD_595_ in a 96 well plate using a Tecan Infinite M200 microtiter plate reader. Measurements were performed every 30 min after addition of ECM proteins for total of 6 hr. Clumping ability was correlated with sedimentation speed. To calculate percent agglutination the following formula was used:
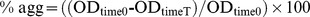



### Light and fluorescent microscopy images of clumps

Microscopy was employed to confirm clumps formed by LAC Δ*arlRS* were distinct from LAC-WT. Briefly, the agglutination assay was performed as described above with either human Fg or Fg-OG. At 2.5 hr post addition of the ECM protein, 25 µL aliquots were removed and spun onto a microscope slide at 400 rpm for 5 min using a Shandon Cytospin 3 (Thermo Scientific Shandon). For light microscopy slides were fixed and stained using the hema-3 stain kit according to manufacturer's instructions (Fisher Scientific, Ca #23-123-869). A Zeiss Axioplan 2 microscope and AxioCam MRm camera (Carl Zeiss Inc.) with Axio Vision 4.1 software was used to capture images of the clumps. For fluorescence microscopy coverslips were added and clumps were assessed by a Zeiss Axioplan 2 microscope and a AxioCam MRn camera (Carl Zeiss Inc.) with Axio vision 4.1 software was used to capture images of the clumps.

### Creation of the *arlRS ebh* mutant strains

Approximately 500 base pairs upstream and downstream of the *arlRS* gene pair (SAUSA300_1307-1308) were amplified using PCR with *S. aureus* strain AH1263 as chromosomal DNA template and the following primer pairs; 1308up5EcoRI and 1308up3fuse; 1308dwn5fuse and 1308dwn3salI. Amplicons were gel purified and joined by PCR using the 1308up5EcoRI and 1308dwn3salI primer pair. The PCR product was gel purified, digested with EcoRI and SalI, and ligated into similarly digested pJB38 [60]. The ligation was transformed into *E. coli* DH5α, selecting on ampicillin and colonies were screened for the correct insert (final plasmid pJMB202). Plasmid pJMB202 was isolated and transformed into RN4220 selecting on TSA containing Cam at 30°C. Plasmid pJMB202 was transduced into AH1263, and single colonies were inoculated into 5 mL of TSB containing Cam. Cultures were grown at 42°C overnight to select for single recombinants. Single colonies were inoculated into 5 mL of TSB medium, grown overnight, and cultures were diluted 1∶25,000 before plating 100 µL on TSA-anhydrotetracycline (150 ng/mL) to screen for loss of pJMB202. Colonies were screened for the double recombination event using PCR with primers 1308verify5 and 1308verify3, and also screened for loss of plasmid by Cam sensitivity.

The Δ*ebh::Kan* mutant was created using the same protocol as outlined above with the following exceptions: 1) the upstream and downstream portions of *ebh* (SAUSA300_1327) were amplified using the following primer pairs: 1327up5EcoRI and 1327up3fuse; 1327dwn5fuse and 1327Dwn3SalI; 2) the 1327up5EcoRI and 1327Dwn3SalI primers were used for joining the two PCR products; and 3) colonies were screened for both Cam sensitivity and Kan resistance after the double recombination event. The 1327 deletion was verified by primer walking. Fourteen sets of primers were designed to amplify 2 Kb regions across the entire *ebh* gene in LAC-WT. The forward primer ebh1for and the reverse primer ebh14rev were used to amplify across the entire gene (33 Kb) for wildtype and 1.8 Kb for the *ebh* deletion. For construction of the Δ*ebh::Tet*, the same approach was used, except that G+tet_nheI and G+tet_mluI oligos were used to amplify the tetM marker.

### Construction of the *arlRS* complement

PCR was used to amplify the *arlRS* gene pair (SAUSA300_1307-1308) and its native promoter using AH1263 chromosomal DNA as a template and the arlcomp5BamHI and arlcomp3SalI primer pair. The PCR product was gel purified and digested overnight with BamHI and SalI. The digested fragment was gel purified, ligated into similarly digested pCM28, and transformed into *E. coli* DH5α cells, resulting in plasmid pJMB219. The constructed plasmid was transformed into RN4220, selected on TSA supplemented with Cam, and subsequently transduced into LACΔ*arlRS*.

### *ebh* promoter fusion studies

A transcriptional promoter fusion with GFP was created using primers JNW47EbhGFPFor and JNW48EbhGFPrev to amplify the region 500 base pairs upstream of the *ebh* predicted start site. The PCR product was gel purified and digested overnight with SphI and BamHI. The gel fragment was cloned into similarly digested pCM11 [61] and transformed into *E. coli* DH5α cells. This construct was transduced into either LAC-WT or LAC Δ*arlRS*. To assess fluorescence, overnight cultures were diluted to a starting OD_600_ of 0.05 in BHI and incubated at 37°C with shaking. Time points were taken periodically by transferring 100 µL to a 96 well plate and measuring the OD_600_ and the fluorescence intensity with excitation at 495 nm and emission at 515 nm using a Tecan Infinite M200 plate reader.

### Quantitative polymerase chain reaction

For qPCR, cultures were grown in BHI to an OD_600_ of 1.5. RNA was isolated using the RNeasy Mini Kit (Qiagen), with the exception that *S. aureus* cells were lysed with 100 µL of lysostaphin for 30 min at 37°C prior to the cell lysis step. Primers were designed for *coa*, *sak*, *vWbp*, *srtA*, *clfA* and *ebh* based on published *S. aureus* sequences using IDT online software (see Supplemental Table S1 for oligonucleotide sequences). Five sets of primers were designed for *ebh* across the entire gene. qPCR was performed by amplifying 10 ng of RNA with Express Super Script Mix (Invitrogen) under the following conditions: 5 minutes at 50°C, 2 minutes at 95°C, 40 cycles of 15 seconds at 95°C and 1 minute at 53°C, followed by a dissociation curve. All samples were run in triplicate and DNA gyrase (*gyrB*) was used as a reference gene. Primer sets one, four and five displayed consistent transcript levels across several experiments and primer set one (SA-ebh-8091 and SA-ebh-7908), located 1/3 of the way into the gene, was chosen as a representative set for all experiments.

### Cloning and purification of the H2 peptide

PCR was performed using oligonucleotides H2 for and H2 rev with Phusion polymerase (New England Biolabs) on AH1263 genomic DNA as template. The PCR fragment was Topo TA (Life Technologies) cloned, transformed into *E. coli* and transformants were screened by PCR. Plasmid DNA from a positive transformant was digested by NdeI and XhoI and ligated into pET28c (Novagen) digested by the same enzymes. The resulting plasmid is called pET28-H2, and the plasmid was transformed into *E. coli* expression ER2566 and saved as AH2930. To purify H2 peptide, cultures of AH2930 were grown at 37°C in LB broth supplemented with Kan to an OD_600_ of 0.5. Isopropyl β-D-thiogalactoside (Research Products International) was added to a final concentration of 0.1 mM, the culture shifted to 30°C and grown for 4 hr. After centrifugation, the pellets were resuspended in equilibration buffer (50 mM sodium phosphate, 0.3 M sodium chloride, 10 mM imidazole, pH 8) with Sigmafast (Sigma) protease inhibitor. Cells were lysed by two passages through a Microfluidics LV1. After centrifugation, the cleared lysate was loaded onto an equilibrated His-Select HF Nickel affinity (Sigma) column. The nonbinding proteins were removed by washing and the 6xHis H2 protein was eluted with the same buffer containing 250 mM imidazole. Fractions containing the H2 protein were pooled, dialyzed versus phosphate buffered saline and concentrated. To address aggregation problems with the protein, it was dialyzed into 8 M urea and purified over an S200 column in 8 M urea. Fractions containing the H2 protein were pooled, dialyzed versus phosphate buffered saline and concentrated in an Amicon Ultra (Millipore) centrifugal filter. This protein was used to generate rabbit polyclonal antisera against H2.

### H2 dot blot

GSSP expression was monitored by dot blotting based on the method described [18]. Strains AH3007, AH3008 and AH3019 were grown in 25 ml of BHI to an OD_600_ of ∼6 and the cell densities were normalized before harvesting. Cells were washed with PBS before resuspending in 10 ml PBS and sonicating for 3 min with a Branson 450 Sonifier (power level 3, 50% duty) to shear off surface proteins. Intact cells were removed by centrifugation and the supernatants were serially diluted (2-fold dilutions) in PBS. Aliquots (5 µL) were spotted on a nitrocellulose membrane and allowed to dry before blocking for 1 h at room temperature with TBS containing 0.05% Tween 20 (TBST) and 5% milk. The membrane was incubated with GSSP H2 antiserum (diluted 1∶1000 in TBST+5% milk) for 1 h, washed three times in TBST, and incubated with HRP-conjugated goat anti-rabbit antibodies (diluted 1∶20,000 in TBST+5% milk). The membrane was washed three times in TBST before incubation with SuperSignal West Pico chemiluminescent substrate for 5 min and exposure to X-ray film.

### Flow cytometry

To assess clumps by flow cytometry, the agglutination assay was performed as described above with the following modifications: fibrinogen conjugated to Oregon Green (Invitrogen, Ca# F7496) was substituted for human Fg, which induced agglutination similarly to fibrinogen as measured by OD. After 2.5 hrs of agglutination, clumps were analyzed on a C6 Accuri flow cytometer and C-flow Plus software using a threshold of 20,000 for data collection and channels FL-1 and FSC-H to measure fluorescence intensity and clump size, respectively. LAC-WT suspended in PBS (negative control) was used to exclude unbound, individual bacteria and cellular debris. Dextran sulfate conjugated to Oregon green (Invitrogen; no clumping control) was used to determine background fluorescence and lack of clumping as dextran sulfate and dextran sulfate conjugated to Oregon green failed to induce agglutination. LAC-WT incubated with Fg-OG was considered 100% agglutination, and the percent agglutination of all strains was set relative to LAC-WT.

### Preparation of clumps for SEM

The agglutination assay was performed as described above with the following strains: LAC-WT, LAC Δ*arl*, LAC Δ*ebh*, LAC Δ*arlRS* complemented strain, and LAC Δ*arlRS* Δ*ebh*. At 2.5 hr post addition of Fg (18.5 µg/mL), 25 µL aliquots of supernate were removed and spun onto a 12 mm round coverslips (Fisherbrand) at 400 rpm for 5 min using a Shandon Cytospin 3 (Thermo Scientific Shandon). Coverslips were transferred to a 24 well plate (Costar) and fixed in 500 µL of 2.5% gluteraldehyde for at least 1 hr. To remove the fix, coverslips were washed three times for 5 min each with a buffer rinse, 0.1 M sodium cacodylate. Samples were then treated with 1% osmium tetroxide for 20 min and washed again with the buffer rinse. The buffer was removed, and samples were washed once in double distilled water and subsequently with increasing concentrations of ethanol for 6 min each to dehydrate the samples. Coverslips were incubated in 95% ethanol for 10 min and 100% ethanol twice for 5 min each. Samples were cross-linked with hexamethyldisilizane (HMDS) twice for 10 min each, HMDS was removed, and samples were allowed to air dry in a tissue culture hood overnight. Coverslips were mounted on stubs and coated with gold particles using an Emitech K550 sputter coater. Images were captured with a Hitachi S-4800 scanning electron microscope with Super ExB filter technology.

### Creation of the *ebh* overexpression strain

Approximately 500 base-pairs upstream of the start codon from the *fabI* gene was amplified using *S. aureus* strain AH1263 chromosomal DNA as a template and the cmR_fabIF and fabI_ebhAR primer pair (see Supplemental Table S1 for sequences). The first 300 base pairs of the *ebh* gene was amplified using *S. aureus* chromosomal DNA as a template and the fabI_ebhAF and Ebh pUC3 primer pair. The Cam^R^ gene was amplified using pJB38 as a template and the yeast_CmF and cmR_fabIR primer pair. A yeast cloning cassette containing the 2μ origin of replication and *Saccharomyces cerevisiae URA3* gene with the native promoter (Boyd JM and Belden WJ unpublished) was amplified using the pUCYeast5 and yeast_CmR reverse primers. The pUC19 plasmid was linearized using AatII and SapI restriction enzymes. All DNA fragments were gel purified and approximately 1 µg of each DNA construct was combined and plasmid circularized with gap repair cloning using *Saccharomyces cerevisiae* creating plasmid JMB449 [62]. The pJMB449 was transformed into in *S. aureus* RN4220 via electroporation, and strains with integrated plasmids were selected for on TSA supplemented with Cam (30 µg/mL). The integrated plasmid was transduced into AH1263 and verified using the 1327 internal verify and cmR_fabIF primer pair. Plasmid integration into AH1263 was verified by DNA sequencing using the 1327 internal verify primer.

### Alpha toxin concentrations

Rabbit blood was purchased from Hemostat Labs. Red blood cell lysis titers to assess alpha-toxin levels were performed as previously described [63].

### Infective endocarditis and sepsis

New Zealand white rabbits (approximately 2–3 kg), either sex, were purchased from Bakkom Rabbitry, Red Wing, MN and used according to University of Iowa IACUC approved protocol 1106138. Rabbits were anesthetized with ketamine (25 mg/kg) and xylazine (25 mg/kg) (Phoenix Pharmaceuticals, Burlingame, CA). Their necks were shaved, and 5 cm incisions were made to expose the left carotid arteries. Hard plastic catheters were inserted into the carotid arteries until the catheters just abutted against the aortic valves. The catheters were then tied in place and allowed to cause damage to the aortic valves for 2 h. Subsequently, the catheters were removed and carotid arteries tied off, and the animals were closed. Animals were injected intravenously through the marginal ear veins with *S. aureus* strains in 2 ml PBS (approximately 10^6^–10^7^ CFU/ml). The rabbits were monitored for health status for up to 4 days; during this time, animals that simultaneously failed to exhibit escape behavior and failed to be able to right themselves, 100% predictive of lethal infection, were prematurely euthanized with 1 ml/kg of Beuthanasia D (Shering-Plough, Westlake, TX). After 4 days (or at the time of premature euthanasia), the animals were euthanized, hearts removed, and vegetation formation determined. Vegetations, cauliflower-like clumps of bacteria and host cells, were removed, weighed, and homogenized for CFU determination. Statistical differences in vegetation weights and CFUs were determined by Student's *t* test analysis of normally-distributed, non-paired data.

### Adherence of bacteria to damaged heart valves

Infective endocarditis was induced in New Zealand white rabbits as described above. Briefly, catheters were inserted into the left carotid artery and allowed to induce damage on the aorta for 2 hrs. Catheters were removed after 2 hrs, the arteries were tied off, and the incision site was closed. Rabbits were infected via marginal ear vein with 2.5×10^7^ CFU. Infection was allowed to proceed for 2 hrs. Hearts were then harvested and washed twice with 30 mLs of sterile PBS to remove bacteria that remained unattached to the heart valve. The leaflets of the aorta were examined and damaged areas were excised. Tissue was homogenized in 1 mL TH media for CFU determination. Statistical differences in CFUs were determined Student's *t*-test analysis of normally-distributed, non-paired data.

## Supporting Information

Table S1 Oligonucleotides used in this study.
(DOC)
