# Supplementary material for: The Staphylococcus aureus ArlRS Two-Component System Is a Novel Regulator of Agglutination and Pathogenesis
Source: PLoS Pathog. 2013 Dec 19;9(12):e1003819. doi: 10.1371/journal.ppat.1003819 (PMC3868527; doi:10.1371/journal.ppat.1003819)
Supplement: Table S1 — Oligonucleotides used in this study. (DOC) [file ppat.1003819.s001.doc]

**Supplemental Table S1.** Oligonucleotides used in this study.

| **Oligonucleotide** | **Sequence** |
| --- | --- |
| qPCR oligos: |  |
| SA-Coa 1080 For | TCCACAGGGCACAATTACAGGTGA |
| SA-sspB 728 F | GTGCAGGATTCAGTATGGCAGCAT |
| SA-sak 350 Rev | GTGACTTCGATCTTTGCGCTTG |
| SA-sak 270 For | TGTCGAATGGGCATTAGATGCGAC |
| SA-vWbp 1136 Rev | TGACGCTCAACTGTAGGCATTGGT |
| SA-vWbp 1035 For | CGACAAGAAACTTGTGGTTTCTGCACC |
| SA-srtA-262U | TATCCAGGACCAGCAACACCTGAA |
| SA-srtA-379D | TCGGACGGTCAATGAAAGTGTGTC |
| SA-clfA-247U | AATAATGGCGAAACGAGTGTGGCG |
| SA-clfA-342D | AGTTACCGGCGTTTCTTCCGTAGT |
| SA-ebh-8091 R | TTGTTGCACTGCTTGCTCTAAGGC |
| SA-ebh-7908 F | TGCGAAGAAGCGTGAAGCAGAAAC |
| SA-ebh-6980 R | GGTTGTGGTGCGACAACTGTGAAA |
| SA-ebh-6867 F | AGCAGGTACTTTCAACCCTGCTGA |
| SA-ebh-17737 R | CTGCAACTGTTGTCGCAACTTCAA |
| SA-ebh- 17625 F | TGTTGCGAACGCTAAGACAACTGC |
| SA-ebh-1368 R | AGGTGTCGCTCCAGCTTCACTAAA |
| SA-ebh-1177 F | GCAGGAGCAGGTGCAAATAAACCA |
| SA-ebh-17559 F | AGACGGTGTCGAAACTGCGTTAGA |
| SA-ebh-17737 R | CTGCAACTGTTGTCGCAACTTCAA |
|  |  |
| Cloning oligos: |  |
| 1327up5EcoRI | GGGGAATTCGGAAGATTAATTCAAGGACTTTCAGCAGCATGTATTATGCCTGC |
| 1327up3fuse | ACGCGTGGTACCGCTAGCCTCCTTATCTTGTTGTTATGTCTCTGTTGTGTTGTGAATG |
| 1327dwn5fuse | GCTAGCGGTACCACGCGTGCCTGTTGAGCGTGTTGATTCGCTTCTTTGTTTTG |
| 1327Dwn3SalI | CCCGTCGACGGGCGTAGAGTTGCAAAAAGAGCAGTTTGGTTAGGC |
| 1327 internal verify | GCAGCCGTTGCCGAATCTTTCTTTGTATTTACATTTTG |
| 1327 verify 5 | GGTAAGATAACTAGCATTGAGAATAAATGGATGGACTACTAAT |
| 1327 verify 3 | CGCGACACTTCCTTTATTACTTCAAAATATTGCAACCTTG |
| 1308up5EcoRI | GGGGAATTCATACATTATGAAATGTTACTTCCAAGTTC |
| 1308up3fuse | ACGCGTGGTACCGCTAGCGCGTCATTTGTACACCTCATATTACGAC |
| 1308dwn5fuse | GCTAGCGGTACCACGCGTCATGACTGAGACGTCAATCAAAGTCATAGGATC |
| 1308dwn3salI | GGGGTCGACCGTTCGGTATTGTTATTAATATGTGTATACTCAAATGC |
| 1308verify5 | CCTAAAGTGTCGTAAGGGTTTACTGC |
| 1308verify3 | CGGTACAAGTGCGTCTACCCCTTC |
| Arlcomp5BamHI | CCCGGATCCGCATCTCCTCGTGTTGATTATTTTGGTTGGCTGACC |
| Arlcomp3SalI | CCCGTCGACGGGAGTACAACAGAAATGATAAAGAACCACTG |
| pCM28veri5 | GCCATTCCAATACAAAACCACATACCTATAATCG |
| pCM28veri3 | GAGCGGATAACAATTTCACACAGGAAACAGC |
| pUCYeast5 | CCGAGCGCAGCGAGTCAGTGAGCGAGGAAGCGGAAGAGCGCGTCAGGTGGCACTTTTCG |
| yeast_CmR | AAGGGTAACTAGCCTCGCCGGTTAGGACCGTTATAGTTACG |
| yeast_CmF | CGTAACTATAACGGTCCTAACCGGCGAGGCTAGTTACCCTT |
| Ebh pUC3 | GGTTCCGCGCACATTTCCCCGAAAAGTGCCACCTGACGTCACGCGTGCTAGCGCGCTACTTTTTGATC |
| cmR_folAR | AAAGATATCTGCGCTACGTTGGAAGCTTGGGTGTGCTTAAATCGGGCC |
| cmR_folAF | GGCCCGATTTAAGCACACCCAAGCTTCCAACGTAGCGCAGATATCTTT |
| folA_ebhAR | CAGCTATTGTTCACTCCTTATGGTACCCATTTCATTTTTTATTACTAA |
| folA_ebhAF | TTAGTAATAAAAAATGAAATGGGTACCATAAGGAGTGAACAATAGCTG |
| G+tet nheI | CCCGCTAGCCGGATTTTATGACCGATGATGAAG |
| G+tet mluI | CCCACGCGTTTAGAAATCCCTTTGAGAATGTTT |
| spa_delA_EcoRI | ATG GAA TTC CAA TCC ACC ATA AAT ACC CTC AA |
| spa_delB | ACG CGT GGT ACC GCT AGC CTT TTT CAA ATT AAT ACC CCC TGT ATG |
| spa_delC | GCT AGC GGT ACC ACG CGT CGC GAA CTA TAA AAA CAA ACA ATA CAC |
| spa_delD SalI | GAT GTC GAC GCT AAA GCG GGA GCA ATT TTC |
| spa_upstream | ATA GCG TGA TTT TGC GGT TT |
| spa_downstream | GCA ACA AAA GAT GTT GCT CGT |
| H2 for | GTTGTT CATATGATGGATGTTAACAC |
| H2 rev | ACTCGAG TTC ACT TGA TTC ATC |
| JNW47EbhGFPFor | aaaaagcttGGTTAAATGCAGGTATGGGA |
| JNW48EbhGFPrev | aaggtaccGCTATTGTTCACTCCTTATCTTG |
